# Supplementary material for: Role and mechanism of NCAPD3 in promoting malignant behaviors in gastric cancer
Source: Front Pharmacol. 2024 Apr 22;15:1341039. doi: 10.3389/fphar.2024.1341039 (PMC11070777; doi:10.3389/fphar.2024.1341039)
Supplement: Supplementary file 11 [file DataSheet2.ZIP › GSEA/Transcipt factor/my_analysis.Gsea.1599461814734/EVI1_04.html]

Details for gene set EVI1\_04[GSEA]

|  || Dataset | filtered\_dataset.sample\_info.cls#WT\_versus\_NCAPD3\_MUT |
| Phenotype | sample\_info.cls#WT\_versus\_NCAPD3\_MUT |
| Upregulated in class | NCAPD3\_MUT |
| GeneSet | EVI1\_04 |
| Enrichment Score (ES) | -0.44546455 |
| Normalized Enrichment Score (NES) | -1.9657495 |
| Nominal p-value | 0.0023980816 |
| FDR q-value | 0.07479509 |
| FWER p-Value | 0.67 |
Table: GSEA Results Summary

  

Fig 1: Enrichment plot: EVI1\_04      
 Profile of the Running ES Score & Positions of GeneSet Members on the Rank Ordered List

  

| SYMBOL | TITLE | RANK IN GENE LIST | RANK METRIC SCORE | RUNNING ES | CORE ENRICHMENT || 1 | 659 | BMPR2 | 16 | 1.047 | 0.0752 | No |
| 2 | 6426 | SRSF1 | 243 | 0.637 | -0.0342 | No |
| 3 | 4281 | MID1 | 294 | 0.595 | -0.0208 | No |
| 4 | 8573 | CASK | 368 | 0.554 | -0.0273 | No |
| 5 | 31 | ACACA | 780 | 0.334 | -0.2947 | No |
| 6 | 8904 | CPNE1 | 814 | 0.308 | -0.2929 | No |
| 7 | 79094 | CHAC1 | 949 | -0.367 | -0.3586 | No |
| 8 | 8609 | KLF7 | 1071 | -0.457 | -0.4076 | Yes |
| 9 | 7168 | TPM1 | 1086 | -0.465 | -0.3791 | Yes |
| 10 | 639 | PRDM1 | 1159 | -0.523 | -0.3874 | Yes |
| 11 | 219790 | RTKN2 | 1204 | -0.579 | -0.3710 | Yes |
| 12 | 1983 | EIF5 | 1219 | -0.595 | -0.3318 | Yes |
| 13 | 3084 | NRG1 | 1320 | -0.739 | -0.3424 | Yes |
| 14 | 57561 | ARRDC3 | 1324 | -0.744 | -0.2828 | Yes |
| 15 | 4622 | MYH4 | 1355 | -0.811 | -0.2372 | Yes |
| 16 | 4734 | NEDD4 | 1369 | -0.856 | -0.1756 | Yes |
| 17 | 26298 | EHF | 1378 | -0.919 | -0.1052 | Yes |
| 18 | 255394 | TCP11L2 | 1408 | -1.538 | 0.0014 | Yes |
Table: GSEA details [plain text format]

  

Fig 2: EVI1\_04      
 Blue-Pink O' Gram in the Space of the Analyzed GeneSet

  

Fig 3: EVI1\_04: Random ES distribution      
 Gene set null distribution of ES for **EVI1\_04**
